# Banana-dependent exercise-induced anaphylaxis

**DOI:** 10.5415/apallergy.0000000000000168

**Published:** 2025-01-13

**Authors:** Öner Özdemir

**Affiliations:** 1 Division of Allergy and Immunology, Department of Pediatrics, Research and Training Hospital of Sakarya, Sakarya University Medical Faculty, Adapazari, Sakarya, Türkiye

***To The Editor:***

I have read the article titled “Preprandial food-dependent exercise-induced anaphylaxis to banana” by Kampitak [1] with great interest. Nevertheless, there are a couple of questions raised in my mind about their case report. And elucidation of these questions in this case report helps the reader understand better of the article.

Food-dependent exercise-induced allergic reactions (FDEIAR) represent a distinctive expression of IgE-mediated food allergy, characterized by the onset of wheals, angioedema, or anaphylaxis following exercise. FDEIAR happen within 6 hours, typically within 2 to 4 hours, of consumption of the culprit food. While the role of exercise and other cofactors in food allergic reactions has already been explored, a comprehensive understanding of these phenomena remains elusive [2, 3].

First, although this case is important because it occurred after a banana consumption, anaphylaxis as described here occurred neither preprandially nor after exercise.

In this case report [1], a 25-year-old man developed gastrointestinal and skin-mucosa symptoms/findings suggestive of anaphylaxis almost immediately after eating 2 bananas. Meanwhile, although the patient had anaphylaxis, it was treated with an antihistamine for the first time and adrenaline was not given. In the intervening period, the patient suffered a second similar episode of anaphylaxis, which resolved after adrenaline was administered. There is no doubt that this was banana-induced and postprandial anaphylaxis. But there is no mention of an exercise component. Again, there is no preprandial food intake as well.

As described in the literature, FDEIAR, in which anaphylaxis occurs after eating postexercise has been reported. I am having difficulty understanding how the author defines this case as “Preprandial food-dependent exercise-induced anaphylaxis to banana.”

Second, in the second paragraph describing the case report [1], there are 2 separate sentences mentioned (He had 2 bananas about 2 hours after exercising in a gym and he had eaten bananas both before and after exercise without incident) that are confusing and question the accuracy of the diagnosis. Guidelines advise FDEIAR patients refrain from consuming the triggering food for 4 to 6 hours before exercise and within 1 to 4 hours following exercise, as well as during exposure to other contributing factors such as aspirin or alcohol [4]. As described in these sentences, anaphylaxis did not recur even though the patient did not avoid food and exercise during the periods when it should have been avoided, casting doubt on the accuracy of the diagnosis.

Third, the fact that the anaphylaxis recurred after a certain period, not a very long period, and after the adrenaline was not administered the first time, makes me think that biphasic anaphylaxis developed instead of FDEIAR [4]. However, this is not mentioned or discussed at all in this case report.

Fourth, FDEIAR diagnosis must rely on patient history and provocation testing, requiring rigorous implementation within a supervised hospital environment [5, 6]. In this case, not only is there a history that casts doubt on the patient’s diagnosis, but the lack of a provocation test with food and exercise to confirm the diagnosis is a major drawback.

In conclusion, I would like to thank the authors for this nice case report and its results. This case contributes to the literature in terms of raising awareness about FDEIAR and showing that banana-induced anaphylaxis may develop. This is a case report that later paved the way for future research FDEIAR on as well.

## Conflicts of interest

The authors have no financial conflicts of interest.

## Author contributions

ÖÖ has done everything.
